# Using a Current Shunt for the Purpose of High-Current Pulse Measurement

**DOI:** 10.3390/s21051835

**Published:** 2021-03-06

**Authors:** Pawel Piekielny, Andrzej Waindok

**Affiliations:** Department of Electrical Engineering and Mechatronics, Faculty of Electrical Engineering, Automatic Control and Informatics, Opole University of Technology, PL-45758 Opole, Poland; p.piekielny@po.edu.pl

**Keywords:** high-current pulses, current measurement, high-current shunts, circuit modeling, Rogowski coil, electrodynamic accelerators

## Abstract

Measurement of high-current pulses is crucial in some special applications, e.g., electrodynamic accelerators (EA) and converters. In such cases, the current shunts have limitations concerning the frequency bandwidth. To overcome the problem, a method based on the shunt mathematical model is proposed. In the method, the solution of ordinary differential equations for the RL circuit is carried out in order to obtain the real current shape. To check the method, as a referee, a Rogowski coil dedicated to measuring high-current pulses was used. Additionally, the measurement results were compared with the mathematical model of the tested power supply system. Measurements were made for the short power supply circuit, which allows eliminating the nonlinearity. The calculations were carried out using a circuit model. In order to obtain the parameters of the shunt (resistance and inductance), it was modeled using an ANSYS/Q3D Extractor software. Comparison of calculation and measurement results confirms the correctness of our method. In order to compare results, the normalized root mean square error (NRMSE) was used.

## 1. Introduction

Design or optimization of electrical devices requires formulation of the correct mathematical model. Thus, an important step in the research is the measurement verification of the obtained results [1,2]. Measurements of excitation current for electrodynamic accelerators and devices powered by short-term current pulses for some milliseconds (or shorter) with an amplitude of kA to even MA are difficult due to the high dynamics of the waveform [3,4]. For current measurements with a peak value of hundreds of kA, current shunts and Rogowski coils are mainly used [5]. However, using shunts for current measurement involves intrusion in the circuit. In cases of measurements in circuits with very low resistance (power supply systems for EA), the ratio of shunt and circuit resistance is relatively high [1].

Measuring shunts enable precise measurement of the peak value and shape of the waveform of relatively fast current pulses. Current shunts are characterized by a wide frequency response, short rise time, and high accuracy of resistance values—they are made in the accuracy class from 0.05 to 0.2. Below the upper frequency limit (−3 dB), the measuring shunts behave like typical DC resistors with a frequency-independent resistance value. Depending on the design used, they may be insensitive to electromagnetic interference. The design of shunts allows the dissipation of relatively high power. The voltage drop across the shunt is proportional to the flowing current. The shunt has a low value of passive parasitic components, i.e., a low value of capacitance and inductance. These parameters are particularly reduced in shunts adapted to alternating currents (ACs). As a result, in practice, capacity and inductance do not affect the quality of measurements in the range from constant signals to signals with a frequency of several kHz [5,6,7,8,9]. For these reasons, manufacturers provide only the resistance value of the shunt. The coaxial shunt resistors are characterized by a relatively large parasitic inductance compared to the wire constructions (which are used in the presented research) [10]. Various mathematical models of current shunts were analyzed and some tests were conducted to determine their full parameters. In [11], the equivalent inductance of the cagelike shunts was measured against a set of four-terminal resistors. In [12], a simulated annealing algorithm was used in order to obtain the LCR parameters, which minimize the mean squared error across different model schemes and real measurements. In both publications, the measurements are the base for the inductance determination. None of the mentioned publications deal with the problem of eliminating the measurement error, arising due to the parasitic inductance of the shunt in the case of high-current pulses. The publications are fixed on the determination of shunt parameters.

There are many different works about Rogowski coils. Current measurements of them can be influenced by many factors ranging from their construction, the shape of the coil loop, and the location of the measured circuit in the coil loop [13,14,15]. Accurate calibration methods have been developed for Rogowski coils dedicated to pulsed power systems [16,17]. Precise calibration allows for obtaining more accurate measurement results. Traditional electromagnetic current sensors cannot accurately measure high-amplitude currents, which is due to the problem of saturation of the magnetic core. In the case of Rogowski coils, this problem does not occur because the flexible core of the coil is made of nonferromagnetic materials, and its turns are evenly wound on this core. Rogowski coils are used for noninvasive current measurement when other measuring methods, such as current shunts or current transformers, are impracticable. Due to their noninvasiveness, Rogowski coils are widely used in diagnostics [18,19,20,21,22]. They are devoted for measuring alternating currents in the range from several hundred mA to several hundred kA and frequencies from tenths of Hz to several MHz. The output voltage from the coil is proportional to the rate of current change [23]. Thus, in our investigations, the Rogowski coil was used as a referee for the calculation results and shunt measurements.

In the presented paper, a comparison of both measuring methods for high-current pulses occurring in electrodynamic accelerators (EA) was carried out. A problem was observed with the shape of a current wave measured by the shunt—there was a relatively high step of current value, which is not typical for an RL load. This step in the current wave is not visible in the model of the system and in measurements using the Rogowski coil. Including the small inductance of the shunt in the mathematical model, the same sharp increase was observed. Based on theoretical and experimental investigations, a method for solving this problem was proposed. It uses a circuit model of the shunt in order to obtain the proper current wave based on the solution of an ordinary differential equation (ODE). The presented method corrects the aberration of the shunt reading caused by its inductance. The correction significantly improves the accuracy of the current wave measurement, which was shown in Section 7 and Section 8.

## 2. Mathematical Model of the Current Shunt

**Figure 1:**
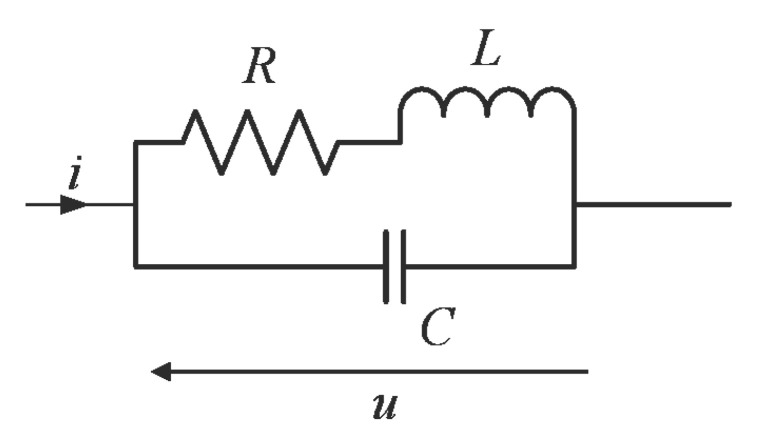
Simplified circuit model of the shunt.

The mathematical model of the investigated current shunt is based on its simplified equivalent circuit presented in Figure 1. The voltage drop on the shunt is determined based on the following ordinary differential equation (ODE):

(1) $$L\frac{di}{dt}+Ri=LC\frac{d^{2}u}{dt^{2}}+RC\frac{du}{dt}+u.$$

The circuit parameters of the model could be determined either by calculation or measurement methods. To determine parasitic parameters by calculations, it is necessary to use electromagnetic field simulation tools, such as ANSYS Maxwell 3D, FEMM, or Opera 3D, which are based on finite element analysis (FEA) techniques to solve the Maxwell differential field equations. However, when the geometry of the test object becomes more complex, full-field simulation is computationally intensive, the models themselves often show poor convergence, and results can be affected by significant errors. The Partial Equivalent Circuit Method (PEEC), which uses Maxwell’s integral equations instead of differential equations and analytically calculates inductance and capacitance based on geometry and material information, is a very common solution [24,25,26]. Such a reduced model can significantly reduce the simulation cost. Therefore, the PEEC method is suitable for simulating high-level objects or in cases where large numbers of design iterations are involved [24,25,26].

Due to the very small value of the shunt passive inductance and capacitance, and due to complex geometry of the current shunt, they were determined using a mathematical model formulated in the ANSYS Q3D Extractor program. It is a tool dedicated for calculating parasitic parameters in any 2D or 3D objects. The software allows calculating RLCG parameters (resistance, inductance, capacity, and conductivity) depending on the given frequency [27]. Q3D Extractor uses the method of moments (integral equations), partial element equivalent circuits (PEEC) method, and finite element method (FEM) to compute capacitive, conductance, inductance, and resistance matrices. It uses the fast multipole method (FMM) to accelerate the solution of integral equations efficiently. The PEEC method requires only geometric and material information. Q3D Extractor computes the full electromagnetic field pattern using the specified mesh and the electrical parameters from the computed field quantities. To include the skin and proximity effects, a nonuniform current distribution is realized by creating a series of layers and gradings based on the skin depth [28].

**Figure 2:**
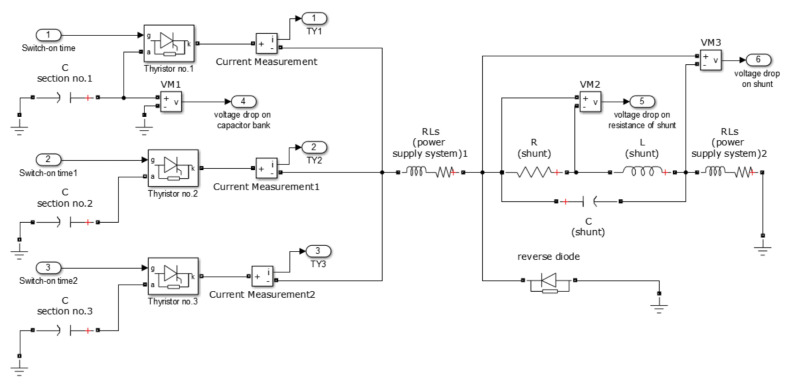
Circuit model of the power supply system and current shunt in Simulink.

For numerical calculations of the power supply system dynamics, the circuit method was used [29,30,31,32]. The circuit model of the shunt was implemented in Matlab/Simulink software. Additionally, in order to compare the calculation results with measurement results, the power supply system was also modeled. The completed circuit model in Simulink is presented in Figure 2.

Due to the relatively small pulse spectrum (less than 1 kHz), the capacitance value was neglected. Thus, the ODE could be written in a more simple form,

(2) $$L\frac{di}{dt}+Ri=u.$$

The knowledge of the shunt parameters allows for recovering the real current waveform based on the Equation (2). Knowing the voltage wave, a current wave could be obtained by integrating the above equation. In our investigations, we implemented a simple Euler method:

(3) $$i_{k+1}=i_{k}+h\cdot \left(u_{k}-\frac{R}{L}i_{k}\right),$$

where

- $i_{k}$—current value in *k*-th step;
- $u_{k}$—voltage value on the shunt at *k*-th step;
- *h*—integration step equal to the sampling time.

Due to its simplicity, the method allows us to determine the real current wave in real-time.

The Rogowski coil was chosen as a referee for current wave measurement. It is characterized by high bandwidth and precision. The measurements were also compared with the calculation results of the shunt model and supply system. In order to compare the measurement results quantitatively, the normalized root mean square error (NRMSE) was used. It is described by the following equation [33]:

(4) $$NRMSE=\frac{\sqrt{\frac{1}{N}\sum_{i=0}^{N}{\left(y_{i}^{meas}-y_{i}^{calc}\right)}^{2}}}{max\left(y_{meas}\right)-min\left(y_{meas}\right)}\cdot 100\%,$$

where

- *N*—number of measurement points;
- $y_{i}^{meas}$—measured value in *i*th point;
- $y_{i}^{calc}$—calculated value in *i*th point.

## 3. Experimental Setup

**Figure 3:**
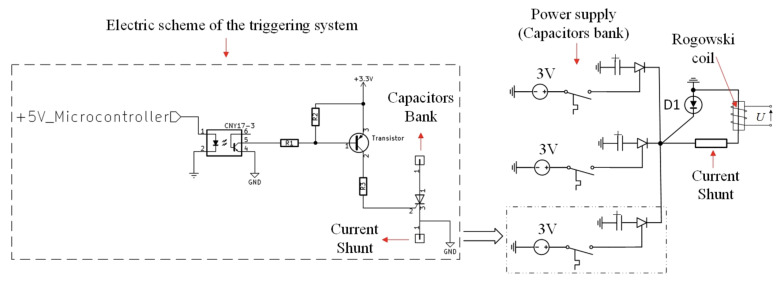
Electrical diagram of the power supply and measurement system.

**Figure 4:**
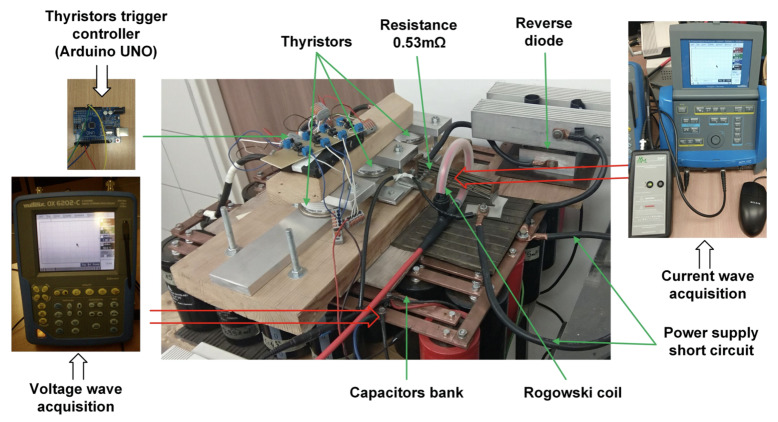
Photographs of the power supply and measurement system.

Comparative analysis of the measurement methods was carried out using the real impulse power supply system of EA. As an energy source, a capacitor bank divided into three symmetric sections with a total capacity of 363 mF was used (Figure 3 and Figure 4).

For switching-on of each section, high-power thyristors were implemented (model T95-1900 from Kubara LAMINA Company) [34]. Both voltage waves on the capacitor bank and current wave were measured using oscilloscopes. The current was measured simultaneously using a Rogowski coil and current shunt. Thus, the comparison of measurement results was possible.

The electric diagram of the test stand is presented in Figure 3. The reverse diode (D1) between the thyristor and the ground of the capacitors’ bank is used to prevent capacitors charging in the negative direction. Due to the high current value, three thyristors were used for switching on. The signals from both measuring systems were recorded on the same oscilloscope. The tests started with switching on the thyristors. The capacitor discharged through the short circuit. The thyristors switched off after discharging of capacitors, and the current flowed through the reverse diode D1.

## 4. Description of the Measuring Current Sensors

### 4.1. Rogowski Coil—Type CWT1500

**Figure 5:**
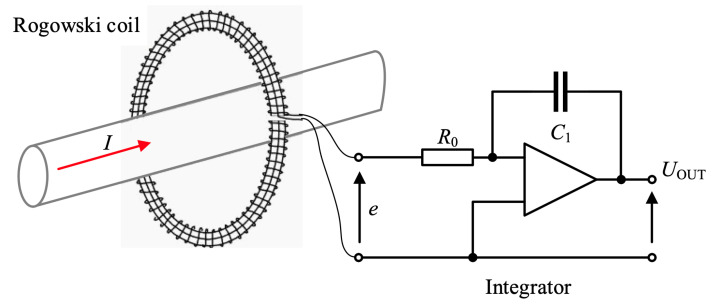
Construction and operation of the Rogowski coil—type CWT1500.

In Figure 5, the construction and principle of operation of the used Rogowski coil with a sensitivity of 0.02 mV/A is presented. The voltage induced in the coil is proportional to the rate of current change within the closed coil circuit. By integrating the voltage from the coil, a voltage value proportional to the current flowing in the circuit inside the coil loop can be obtained. The measurement error of the coil depends on the location of the measured circuit in the coil loop. The declared relative error for the central location of the measured circuit is in the order of 0.5%. Along with the change of the position of the perimeter regarding the edge of the coil loop, the measurement error increases. The loop does not need to be circular [23].

The induced voltage in a closed measuring coil is described by the following relationship:

(5) $$e=\mu_{0}NA\frac{di}{dt}=H\frac{di}{dt},$$

where

- H(Vs/A)—coil sensitivity;
- i(A)—measured current in the circuit passing through the loop of coil;
- *N*—number of turns of the coil;
- $A(m^{2})$—cross-sectional area of the coil core.

The output voltage $U_{out}$ is described by the expression

(6) $$U_{out}=\frac{1}{T_{i}}\int edt=R_{SH}I,$$

where

- $T_{i}=R_{0}C_{1}$—integrator time constant;
- $R_{SH}=\frac{H}{T_{i}}$—Rogowski coil sensitivity.

A dedicated data acquisition system was used in order to measure the voltage in the coil. The oscilloscope acquired the output voltage signal. Table 1 contains the most important parameters of the Rogowski coil used. The length of the coil loop is 300 mm. The converter has an output voltage of 6 V for the rated peak current of 300 kA. If the peak current exceeds this value, the integrator saturates and the measured waveform is incorrect (unlike an amplifier for which the output waveform is cut off). The Rogowski coil is connected to the integrator by a coaxial cable, being an inseparable product of the company Power Electronic Measurements Ltd. [35].

### 4.2. Current Shunt

**Figure 6:**
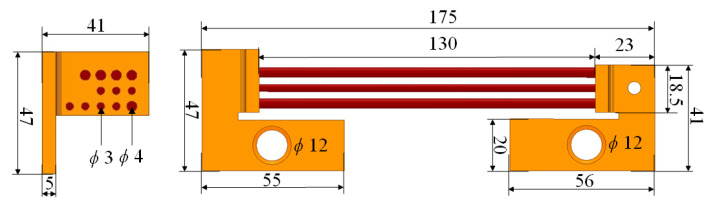
Outline of the investigated current shunt (dimensions in mm).

In Figure 6, the outline of the current shunt of accuracy 0.1 was presented. The shunt used in conjunction with the oscilloscope enables recording of impulses and shock current waveforms with values of the order of several hundred kA.

The current shunt is made of copper mounting bases and thrust rods. The following parameters were adopted for the calculations:

- copper conductivity $\sigma =58\cdot 10^{6}$ (S/m);
- rods’ conductivity $\sigma =23.2\cdot 10^{4}$ (S/m).

The conductivity of rods is approximately 200 times lower compared with copper.

**Figure 7:**
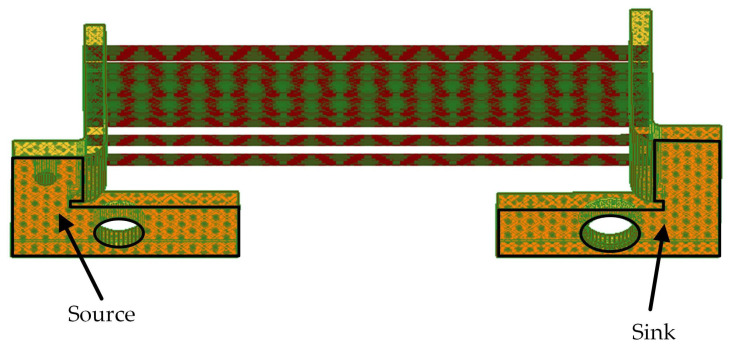
A Q3D model.

The numerical model in the Q3D software is presented in Figure 7. The boundary conditions, i.e., the source and sink were assumed on the bottom part of the terminals. In the investigated case, the pulse spectrum occupation is up to f=1 (kHz). Thus, the skin depth of shunt rods is equal to

(7) $$\delta =\frac{1}{\sqrt{\pi \cdot f\cdot \mu \cdot \sigma }}=\frac{1}{\sqrt{\pi \cdot 1000\cdot 4\pi \cdot 10^{-7}\cdot 23.2\cdot 10^{4}}}=33\;\left(\mathrm{mm}\right)$$

and does not influence the calculation results significantly (the rods’ diameters do not exceed 4 (mm)). Although, the skin depth of copper is lower (2.1 (mm)), as the numerical tests show, thus influencing the current density distribution only weakly.

**Figure 8:**
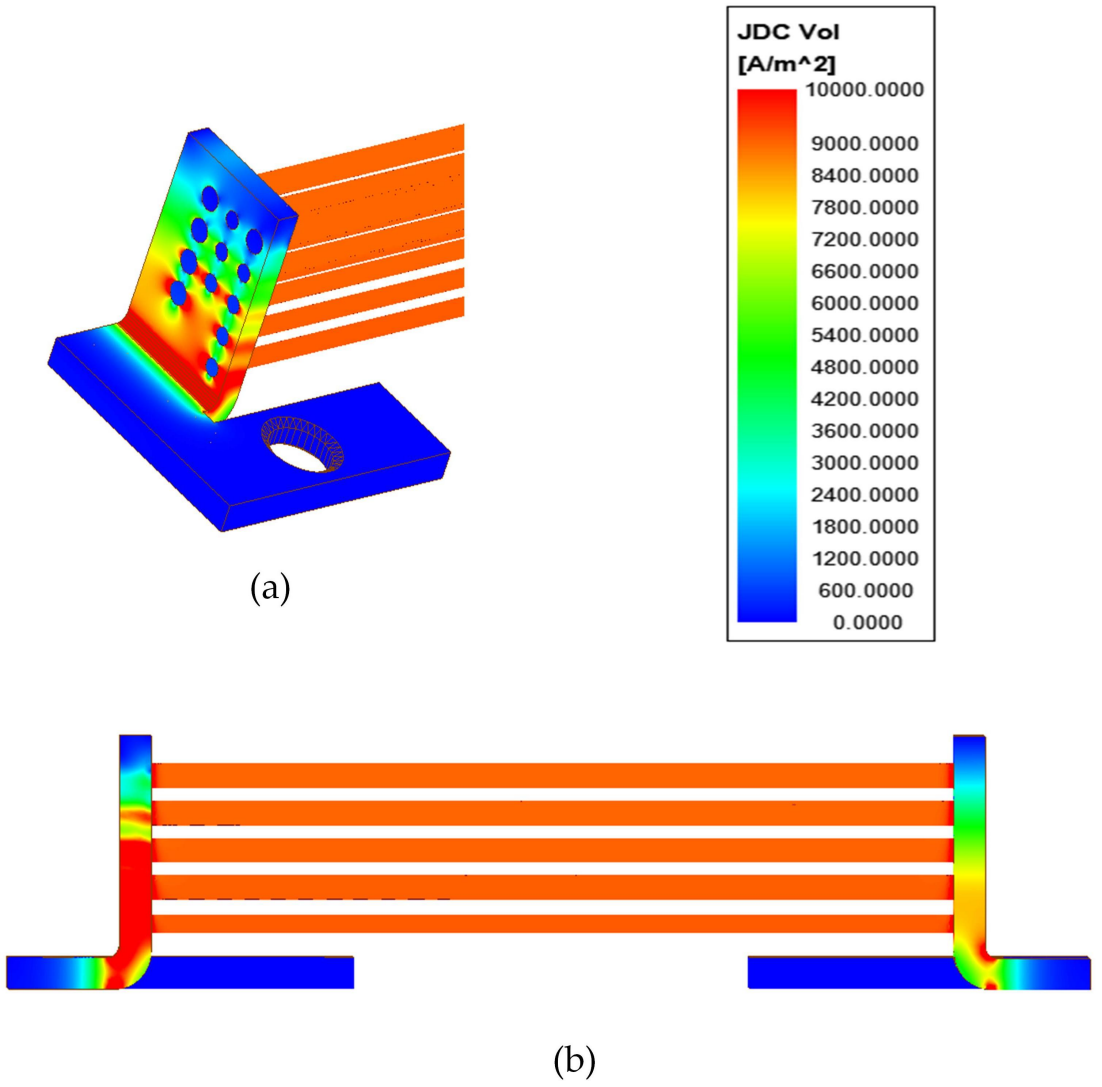
Current density distribution in the shunt: (**a**) front part of one terminal, (**b**) side view.

**Figure 9:**
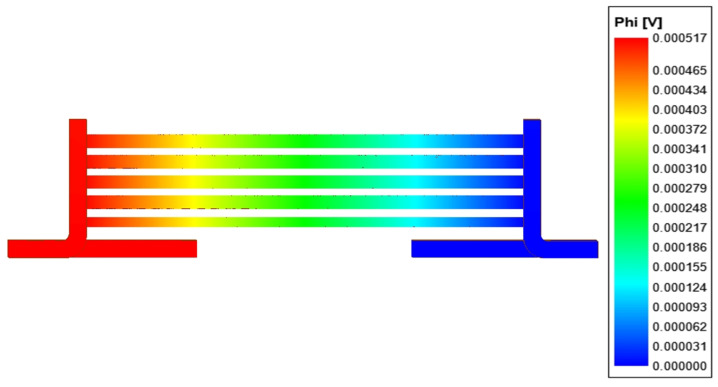
Potential distribution in the investigated shunt.

In rods of the investigated current shunt, the current density distribution is homogenous (Figure 8b). In the terminals, the highest value is observed in the curved part (Figure 8a). The current density value decreases along with the distance from curve. It is due to the fact that the total current flowing in the terminal is divided into the rod currents. The electric potential decreases approximately linearly along the shunt (Figure 9), which is due to linear conductors. Results of the shunt circuit parameters calculation, made for the DC case, are presented in Table 2.

The calculated shunt resistance was verified experimentally. A value of 0.53 mΩ was obtained, which is very close to the theoretical one. Measurements were carried out using a 24-bit measurement card NI9219 with a measurement range from −125 mV to 125 mV (the measurement resolution of the card was 7.45 nV). The current was measured using a current transducer LEM with a rated measuring current of $I_{n}=50$ A. In addition, the shunt inductance value was determined experimentally and a 91.59 nH value was obtained. The value is 7.12 nH higher than the result of numerical calculations. However, the discrepancy is less than 10%. Both experimental and numerical results confirm that the value of the current shunt inductance is an important factor influencing the measurement of high-current pulses.

## 5. Comparison of Measurement Results Obtained from Rogowski Coil and Current Shunt

**Figure 10:**
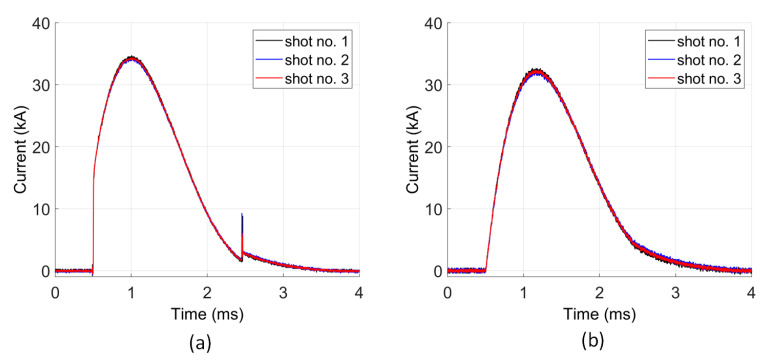
Measurement repeatability of current waves for U=98 V: (**a**) current shunt; (**b**) Rogowski coil.

In the first step to compare the waveforms from the Rogowski coil and the current shunt, measurement repeatability was tested. In Figure 10, the waveforms of the currents from the Rogowski coil (Figure 10b) and current shunt (Figure 10a) are presented. The triggering of the impulse supply system was set to be the same for all waves. There are only very small differences in the recorded waveforms observed, which result directly from the fluctuation of the pulse trigger voltage. The RMS error is equal to 0.255 kA in case of the shunt and 0.315 kA in case of the Rogowski coil measurements, which is less than 1% of the maximum current value. This means that the waveforms obtained from both measurement methods are repeatable. Thus, a comparison of them could be made.

**Figure 11:**
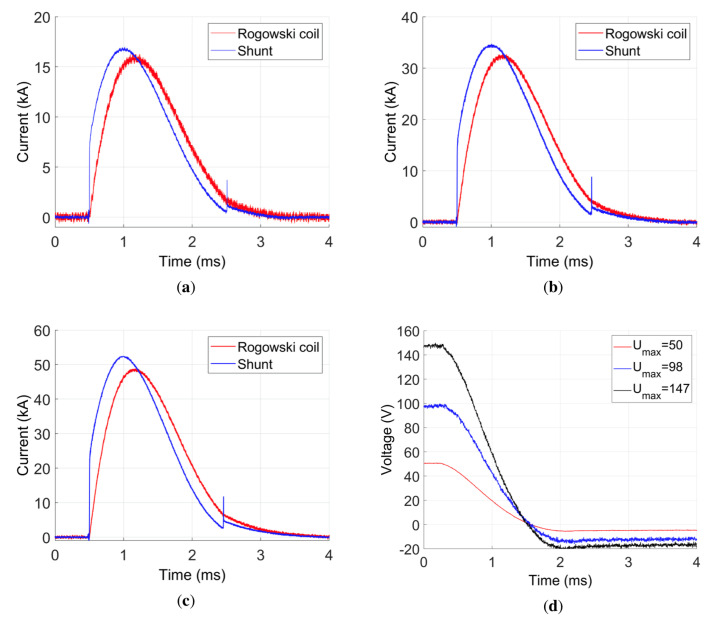
Comparison of power supply waves: (**a**) excitation current vs. time for U=50 V; (**b**) excitation current vs. time for U=98 V; (**c**) excitation current vs. time for U=147 V; (**d**) voltage waves for all initial voltage values.

In Figure 11a–c, a comparison of current waveforms obtained from the Rogowski coil and the current shunt measurements for different values of the initial supply voltage is presented. Independent of the voltage value, the current waveforms for a given measuring method have the same shape—they differ only in the maximum value, which results directly from the initial supply voltage.

The measured current waveforms differ depending on the measurement method used. In the case of a current shunt, there is a more rapid slope of current increase, and hence higher amplitudes in relation to the Rogowski coil were observed. The short pulse, observed in the shunt measurement at 2.5 ms, is arising due the voltage pulse generated on its inductance due to the rapid current change. It does not cause any current flow, thus, it is not visible in the Rogowski coil measurement. The measurement using a current shunt is more sensitive to overvoltages associated with the commutation of semiconductor elements of the power supply system. Due to the finite inductance value of the circuit, the current wave measured by the current shunt is not proper. The current cannot increase abruptly. The problem arises due to the nonzero parasitic inductance of the shunt (Table 2).

In Figure 11d, the waveforms of voltage drop on the capacitor battery for the subsequent series of measurements are presented. The capacitor bank is negatively charged, despite the reverse diode. The negative voltage value is higher for higher current pulses. It is due to the finite reverse time of the diode. The voltage waves cross the zero value at the same time: 1.55 ms. This means that the discharge time does not depend on the supply voltage.

The values of NRMSE for the waveforms presented in Figure 11 are given in Table 3. There is a significant error value observed (approximately 11%). The value increases along with the initial voltage value. In the case of a railgun, the driving force depends directly on the current value. Thus, in order to investigate such a device, it is important to determine the real current wave. In case of the shunt, the measurement error is too large to be credible.

## 6. Measurement Verification of the Current Shunt Mathematical Model

The mathematical model presented in chapter 2 was verified experimentally. In Table 4 and Table 5, measured parameters of the power supply system and thyristors are presented.

**Figure 12:**
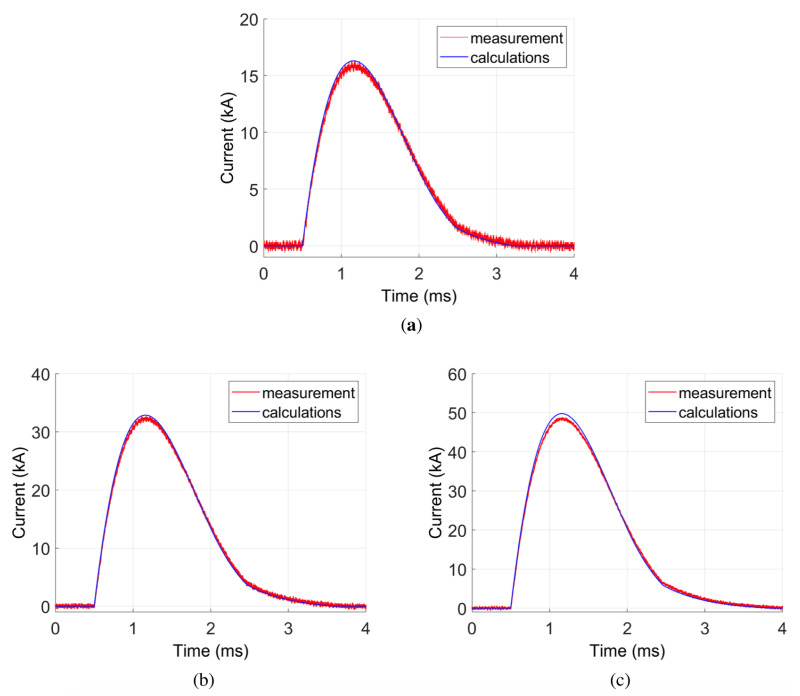
Measurement verification of the model using the Rogowski Coil for different initial voltage values: (**a**) U=50 V; (**b**) U=98 V; (**c**) U=147 V.

In Figure 12, the comparison between Rogowski coil measurement and the calculation model for the current wave is presented. Different values of initial capacitor voltage were assumed: 50 V, 98 V, and 147 V. Remaining initial conditions were assumed to be zero. A good conformance is observed. Some differences occur, mainly for the amplitude of the measured signal. The NRMSE error between measurements and calculations is between 1.16% and 1.68% (Table 6), which is a low value. This proves the quality of the calculation model and confirms that the Rogowski coil measures the real current wave in the system. The differences are mainly due to some simplification of the mathematical model, e.g., the eddy currents in the wires were neglected. Additionally, the measured system parameters could slightly differ from the real ones, which influences the calculation results.

**Figure 13:**
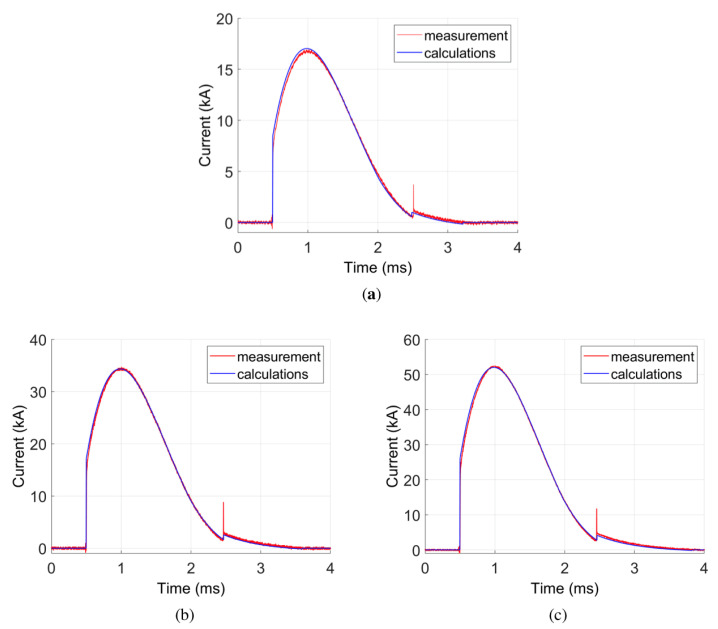
Measurement verification of the shunt circuit model for different initial voltage values: (**a**) U=50 V; (**b**) U=98 V; (**c**) U=147 V.

A similar comparison between the model and measurement results is presented for the current shunt (Figure 13). In this case, the voltage waves (voltage drop on the shunt) obtained both from calculations and measurements were converted to current waves. In this test, the current shunt resistance value R was determined experimentally, while the inductance value L was chosen to best fit the current shape in calculation and measurement results (Table 7). The resistance and inductance values are very close to that obtained by the ANSYS Q3D Extractor model (Table 2), which confirms its correctness. The capacitance was assumed equal to zero, since it does not influence the results significantly.

Taking into account the shunt inductance, a very good agreement between calculations and measurement results was obtained (Figure 13). The NRMSE error between current waves obtained from the mathematical and physical model of the shunt is in between 1.39% and 1.71% (Table 8). The error value is slightly higher than for the Rogowski coil model, which may be due to the sensitivity of the method to overvoltages. The results confirm correctness of the transient mathematical model. Slight differences between measurements and calculations are, similarly as in the case of Rogowski coil, due to some simplification of the mathematical model.

Comparing current waves obtained from the Rogowski coil and current shunt, a significant difference is visible (Figure 12 and Figure 13). Based on the mathematical model, it is assumed that the Rogowski coil measures the real current waveform in the system. In the case of high-current peaks, the current shunt does not give the proper current wave shape. This is due to its parasitic inductance. Although it is relatively small (80 nH), in the case of high-current pulses, it influences results considerably, which is visible both in measurements and numerical models. Two current steps observed in the current wave (at 0.5 ms and 2.5 ms) are due to this inductance. The first step at 0.5 ms occurs after switching on the circuit (a voltage is induced according to the electromotive force e=Ldi/dt). The second step is due to diode commutation. The electromotive force induced on the shunt inductance practically does not cause any current flow in the circuit. Thus, these peaks are not visible in the Rogowski coil measurement.

## 7. Recovering a Real Current Waveform from Shunt Voltage Measurement

**Figure 14:**
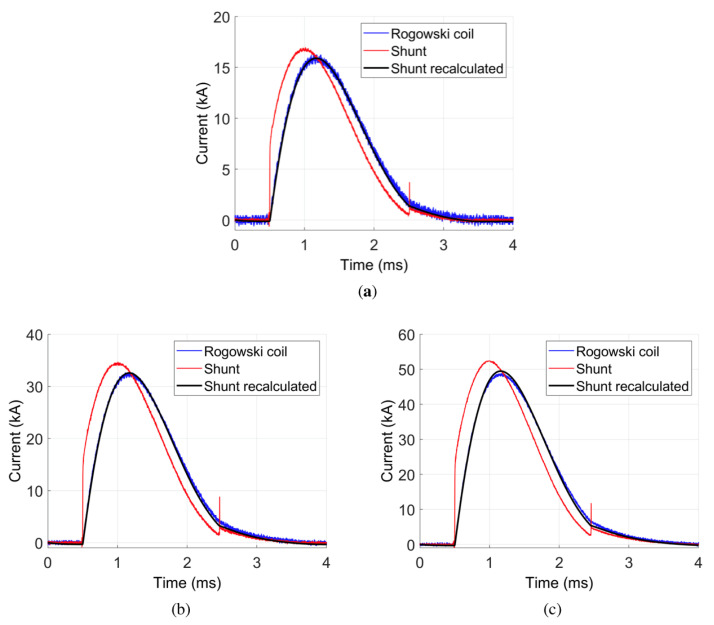
Comparison of different current waveform measurements: (**a**) U=50 V; (**b**) U=98 V; (**c**) U=147 V.

The expression (3) for recovering the current waveform is very simple and could be implemented in real-time applications. In the presented investigations, it was implemented in Simulink software. As the input, the voltage wave measured on the current shunt was used. The results obtained using the expression (3) are shown in Figure 14 (black line, Shunt recalculated). For lower voltage values (below 100 V, Figure 14a,b), there is almost no visible difference between the current waves obtained from Rogowski coil and from shunt after applying Equation (3). For higher voltages, only a slight difference is observed (Figure 14c), which could be due to errors in shunt parameters determination. In Table 9, current peak values measured by investigated methods are given.

The Rogowski coil measurement was assumed as a referee. The values obtained from the shunt after implementing our method are characterized by a significantly lower error than those obtained directly from the voltage drop measurement (red line, Shunt). The NRMSE error for waveforms obtained after applying Equation (3) is between 1.15% and 1.35% (Table 10). The error is relatively low, which satisfies the presented method. Thus, knowing all shunt parameters allows for obtaining the correct wave of the current, even for high-value current pulses.

The most important advantage of the presented method is its simplicity. The only problem is determining the inductance of the current shunt (the resistance is known). This could be done either by measurements or by magnetostatic field calculations using the finite element method (FEM). In the case of the field modeling, all dimensions of the shunt and all material properties must be known.

## 8. Tests for a Second Current Shunt

**Figure 15:**
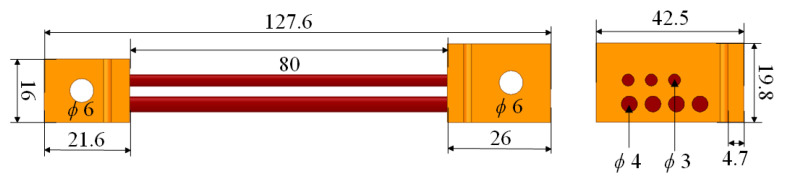
Outline of the second current shunt (dimensions in mm).

**Figure 16:**
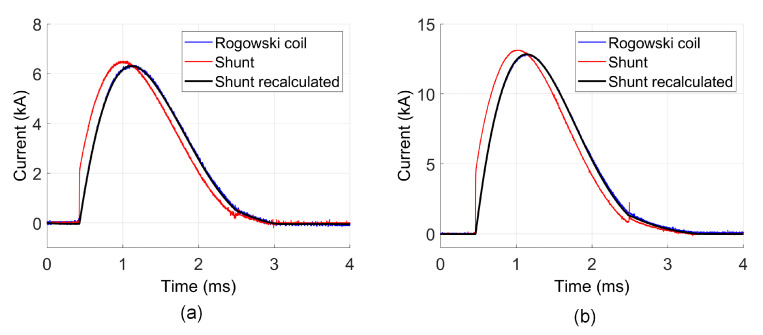
Comparison of different current waveform measurements: (**a**) U=20 V; (**b**) U=40 V.

In order to test the proposed method more deeply, calculations and measurements for a shunt, which differs from the previous one, were carried out. The outline and dimensions of it are presented in Figure 15. Compared to first one, the shunt is characterized by a lower number of rods and smaller length. In Table 11, the parameters of the shunt obtained from Q3D model are given. These parameters were used to recover the original current waveform. The results are presented in Figure 16. Due to the much lower nominal current of the shunt, the tests were made for lower voltage values.

Similar to the previous measurement, the Rogowski coil results were assumed as a referee. The errors values are given in Table 12 and Table 13. The current peak values differ more than 2.5% between Rogowski coil and shunt measurement results. After recalculation, the differences are less than 1%. The NRMSE value decreases after correction from more than 7.5% to below 1%, which is even lower than in the tests shown in chapter 7. Thus, the presented method significantly reduces the measurement error for the current shunt.

## 9. Conclusions

For a high-current pulse measurement (some kA) there are very significant differences between current waveforms obtained from a shunt and visible Rogowski coil. The proper current wave is measured by the Rogowski coil, while the shunt does not give the correct result. Both mathematical models and measurements show that this is mainly due to the inductance of the shunt. The value obtained from measurements (91.59 nH) is slightly higher than that obtained from the magnetostatic field model (7.8%) and from synthesis using a circuit model (12.6%). Although the value is very small, it needs to be taken into account in the current peak measurements. The capacitance of the shunt does not influence the calculation results significantly.

From the knowledge of the shunt parameters, it is possible to recover the real current waveform. A simple Euler method used for integration of an ODE describing the RL element is sufficient to significantly improve the accuracy, which was presented for two different current shunts. The simplicity of the method allows using it in real-time calculations. Thus, the shunt could be used in the measurements of high dynamic current waves (e.g., in railguns, magnetizing devices) after applying the correction arising from Equation (3). Taking into account the higher price of Rogowski coil, in the case of some measurements, it is more convenient to use a current shunt including the presented correction method.

In order to explain problems with the measuring of high-current pulses using a current shunt, we used both numerical models and experimental investigations. The most difficult task was choosing an appropriate model of the shunt. In the presented case, the Q3D software was successfully implemented. The presented approach is very useful and not only allows us to explain problems with current pulses measurement, but also indicates the way to solve them.

## Figures and Tables

**Table 1:** Parameters of the used Rogowski coil—type CWT1500.

Sensitivity	Peak Current	Peak *di*/*dt*	Droop Typ.	LF (3 dB) Bandwidth	HF (3 dB) Bandwidth
(mV/A)	(kA)	(kA/μs)	(%ms)	(Hz)	(MHz)
0.02	300	40	0.035	0.03	16

**Table 2:** Shunt circuit parameters—results of numerical calculations.

R (mΩ)	C (pF)	L (nH)
0.527	4.73	84.47

**Table 3:** Normalized root mean square error (NRMSE) values for transients given in Figure 11.

Performance Graph	Analyzed Wave	NRMSE (%)
Figure 11a	i(t)—for U=50 V	10.67
Figure 11b	i(t)—for U=98 V	11.17
Figure 11c	i(t)—for U=147 V	11.22

**Table 4:** Power supply parameters (excluding shunt).

Element	Rs (mΩ)	C$s_{\mathrm{total}}$ (mF)	Ls (μH)
Basic elements	1.08	363.0	78.0

**Table 5:** Thyristor parameters adopted.

Element	Rs (mΩ)	Lon (nH)	Vf (V)	Il (A)	Tq (μs)
Thyristors	0.16	50	0.8	0.1	100

**Table 6:** NRMSE values for transients given in Figure 12.

Case Number	Performance Graph	Wave	NRMSE (%)
1	Figure 12a	i(t)—for U=50 V	1.68
2	Figure 12b	i(t)—for U=98 V	1.16
3	Figure 12c	i(t)—for U=147 V	1.43

**Table 7:** Assumed current shunt parameters.

R (mΩ)	C (pF)	L (nH)
0.53	0	80.0

**Table 8:** NRMSE values for transients given in Figure 13.

Case Number	Performance Graph	Wave	NRMSE (%)
1	Figure 13a	i(t)—for U=50 V	1.71
2	Figure 13b	i(t)—for U=98 V	1.39
3	Figure 13c	i(t)—for U=147 V	1.44

**Table 9:** Measured current peak values.

U(V)	Rogowski Coil (kA)	Shunt (kA)	Error (%) vs. Rogowski Coil	Shunt Recalculated (kA)	Error (%) vs. Rogowski Coil
50	16.50	16.95	2.73	16.40	0.61
98	32.90	34.72	5.53	33.07	0.52
147	49.05	52.64	7.32	50.08	2.10

**Table 10:** NRMSE value for transients given in Figure 14 (shunt recalculated).

Case Number	Performance Graph	Wave	NRMSE [%]
1	Figure 14a	i(t)—for U=50 V	1.35
2	Figure 14b	i(t)—for U=98 V	1.24
3	Figure 14c	i(t)—for U=147 V	1.15

**Table 11:** Second shunt circuit parameters—numerical calculations results (Q3D).

R (mΩ)	L (nH)
0.516	54.74

**Table 12:** Measured current peak values.

U (V)	Rogowski Coil (kA)	Shunt (kA)	Error (%) vs. Rogowski Coil	Shunt Recalculated (kA)	Error (%) vs. Rogowski Coil
20	6.35	6.51	2.52	6.30	0.79
40	12.89	13.15	2.02	12.82	0.54

**Table 13:** NRMSE value for transients given in Figure 16.

Case Number	Performance Graph	Wave	NRMSE (%) Shunt	NRMSE (%) Shunt Recalculated
1	Figure 16a	i(t)—for U=20 V	7.56	0.72
2	Figure 16b	i(t)—for U=40 V	8.17	0.78
